# Introduction of a new model for time-continuous and non-contact investigations of in-vitro thrombolysis under physiological flow conditions

**DOI:** 10.1186/1471-2377-11-58

**Published:** 2011-05-26

**Authors:** Florian C Roessler, Marcus Ohlrich, Jan H Marxsen, Marc Schmieger, Peter-Karl Weber, Florian Stellmacher, Peter Trillenberg, Jürgen Eggers, Günter Seidel

**Affiliations:** 1 Department of Neurology, University Hospital of Schleswig-Holstein, Campus Lübeck, Ratzeburger Allee 160, 23538 Lübeck, Germany; 2 Department of Internal Medicine, Haematology, University Hospital of Schleswig-Holstein, Campus Lübeck, Ratzeburger Allee 160, 23538 Lübeck, Germany; 3 Fraunhofer Institute for Biomedical Engineering (IBMT), Ultrasound Systems Development, Ensheimer Str. 48, 66386 St. Ingbert, Germany; 4 Research Centre Borstel, Clinical and Experimental Pathology, Parkallee 3a, 23845 Borstel, Germany; 5 Department of Neurology, Asklepios Klinik Nord, Langstedter Landstr. 400, 22417 Hamburg, Germany

## Abstract

**Background:**

Thrombolysis is a dynamic and time-dependent process influenced by the haemodynamic conditions. Currently there is no model that allows for time-continuous, non-contact measurements under physiological flow conditions. The aim of this work was to introduce such a model.

**Methods:**

The model is based on a computer-controlled pump providing variable constant or pulsatile flows in a tube system filled with blood substitute. Clots can be fixed in a custom-built clot carrier within the tube system. The pressure decline at the clot carrier is measured as a novel way to measure lysis of the clot. With different experiments the hydrodynamic properties and reliability of the model were analyzed. Finally, the lysis rate of clots generated from human platelet rich plasma (PRP) was measured during a one hour combined application of diagnostic ultrasound (2 MHz, 0.179 W/cm^2^) and a thrombolytic agent (rt-PA) as it is commonly used for clinical sonothrombolysis treatments.

**Results:**

All hydrodynamic parameters can be adjusted and measured with high accuracy. First experiments with sonothrombolysis demonstrated the feasibility of the model despite low lysis rates.

**Conclusions:**

The model allows to adjust accurately all hydrodynamic parameters affecting thrombolysis under physiological flow conditions and for non-contact, time-continuous measurements. Low lysis rates of first sonothrombolysis experiments are primarily attributable to the high stability of the used PRP-clots.

## Background

In ischemic stroke therapy, a critical need exists for early treatment to prevent neuronal loss. Several trials have shown that thrombolytic therapy in cerebral ischemia can reduce stroke disability in selected patients. In addition, the increased use of thrombolytic therapy has revealed significant limitations and stimulated efforts to improve effectiveness and decrease bleeding complications.

The use of ultrasound to enhance thrombolytic therapy (called sonothrombolysis) represents a complementary approach with unique features, as its effects are limited to the insonified area. Therefore, thrombolytic activity can be enhanced at the site of the vessel occlusion without increasing the risk of systemic bleeding complications. Although the supporting effect of ultrasound for thrombolysis is proven [1-4] and an enhanced lysis rate by a combined exposure to ultrasound and thrombolytics [5-7] or gas-filled microbubbles has been demonstrated [8,9], the underlying mechanisms are still unknown and require further investigations. In-vitro models are most suitable for such basic research, because they are safe, facilitate fast and accurate adjustments of experimental parameters, are inexpensive compared to animal or clinical studies, and provide reproducible results.

Most in-vitro models study the effect of thrombolytics or ultrasound on a clot stored in a resting fluid by measuring its weight loss [6,7,10-13] or the wash-out of radioactivity when radiolabelled clots are used [14,15]. Other experimental setups provide clots within fluids with a constant flow velocity [4,16-19]. Reperfusion is measured here by collecting the perfusion fluid per minute [16], by using photoelectric techniques [19], or by detecting flow velocities [18]. Clot dissolution is measured by weighing [4,17] or detecting concentrations of radiolabelled clot components [18]. A few investigators have applied roller pumps to generate pulsatile but unphysiological flow [20]. It has been frequently pointed out, however, that physiological flow and pressure have to be considered in experiments for thrombolysis, because the mechanical stress of streaming fluids affects clot lysis [21-25]. Knowledge about the interaction of thrombolysis and haemodynamic parameters such as blood pressure might lead to far-reaching consequences for clinical treatment [25-27].

Only a few models allow temporally resolved measurements [18,19]. Time-continuous measurements of the temporal dynamic of thrombolysis, however, are essential in defining a lysis strategy that is both effective and safe. In most models, the clot has to be removed from the experimental setup to determine the lysis rate when using a method such as weighing. Methods like this interfere with the process of thrombolysis and might lead to inaccuracies.

In sight of these considerations the aim of this work was (1) to introduce a new in-vitro pulsatile flow model that meets these conditions, (2) to prove its reliability and describe its characteristics, and (3) to demonstrate its feasibility by first experiments.

## Methods

**Figure 1:**
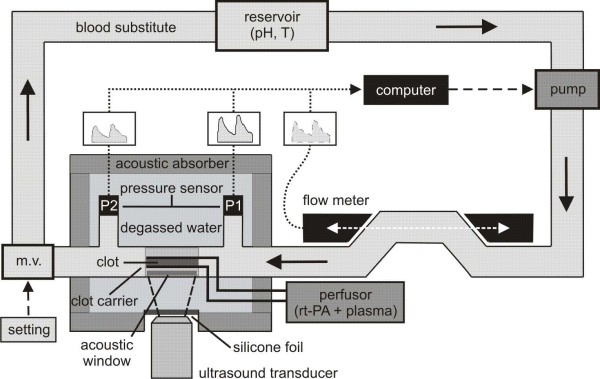
**Model setup**. Detailed explanations are given in the text. m.v. = modulating valve. Solid arrows: Direction of the streaming blood substitute. Dashed arrows: Options to modify hydrodynamic conditions. Dotted arrows: Acquisition of data.

**Figure 2:**
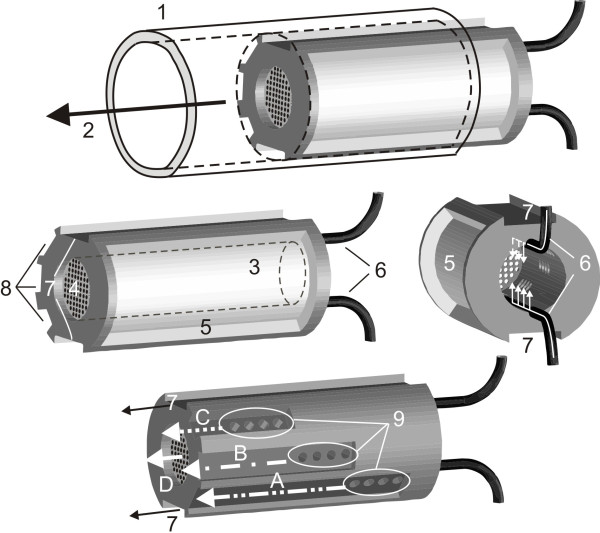
**Custom-built clot carrier**. Detailed explanations are given in the text. (1) tube system, (2) direction of streaming blood substitute, (3) cavity for the clot, (4) gauze, (5) gap for ultrasound application, (6) tubes for thrombolytic supply, (7) channels reducing the hydrodynamic resistance of the carrier, (8) channels connected with the clot cavity by small holes (9) providing an decreased hydrodynamic resistance when the clot is getting smaller. White arrows A - D: Successively increased flow rate during clot dissolution.

The experimental setup is shown in Figure 1. A computer-controlled pump generates pulsatile flow velocities at defined values. A blood substitute (Ringer solution, Berlin-Chemie Menarini, Berlin, Germany), stored in a reservoir at a controlled temperature of 37°C and a pH of 7.4, circulates through a tube system (sheathed PVC, 6 mm inner diameter). Varying the computer-controlled flow velocity and the resistance of the tube system using a modulating valve (Teflon^® ^1/8'' FNPT: Aalborg, Orangeburg NY, USA) leads to a defined adjustment of pressure and flow velocity at the same time. A clot is placed within a custom-built carrier, permitting accessibility for thrombolytics and ultrasound. The part of tube system containing the clot carrier runs through a basin filled with degassed water. Ultrasound is applied through a flexible silicone foil and enters the inside of the clot carrier through an acoustic window. Acoustic absorbers prevent ultrasonic reflections. Thrombolytics are applied at continuous rates via small tubes into the interior of the clot carrier (see also Figure 2). One flow velocity sensor before and two pressure sensors located 0.07 m before and behind the clot carrier are used to record data online. Because of its special shape, the pressure decline at the clot carrier is monotonically related to the hydrodynamic resistance of the clot and, therefore, to its size. We aim to detect reductions in this pressure decline as a new means of measuring the lysis rate of the clot.

### Computer-controlled pump

**Figure 3:**
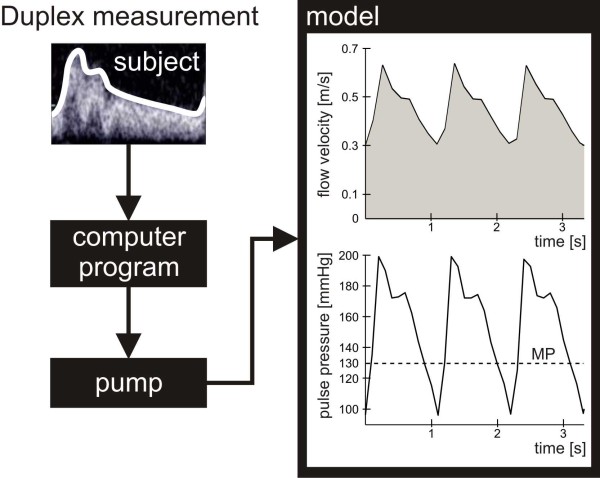
**Creation of physiological flow velocity profiles**. The envelope-data of flow velocity profiles obtained by duplex measurements in normal internal carotid arteries of healthy subjects are supplied to a custom-built computer program controlling a progressing cavity pump that excites a replication of the measured flows in the tube model. Thereby and by adjusting the modulating valve, for the first lysis experiments flow velocity curves and pulse pressure waves of a fictitious patient suffering from hypertension were generated. MP = mean pressure.

A progressing cavity pump (2-E15A: Netzsch, Selb, Germany) was used. With a custom-built program (LabVIEW) controlling the pump, any given pulsatile or constant flow velocity can be achieved within a range of 0 to 0.95 m/s. The pulsatile flow used in the experiments reflects common physiological flow velocity curves found in the internal carotid artery of healthy subjects recorded by duplex measurements (Figure 3).

### Clot carrier

Figure 2 shows the design of the custom-built clot carrier. During the experiments, the carrier is located inside the tube system filled with the blood substitute. The carrier is composed of a cylindrical pipe made of polyoxymethylene (POM) with a concentrical cavity where the clot is placed during the experiments.

The cavity is closed at the distal end by gauze. A gap at one side of the pipe allows the application of ultrasound with a minimized loss of energy. Using small tubes, human plasma and thrombolytics can be applied directly into the cavity of the pipe where the clot is located. Perfusor syringes (Pilot A2, Fresenius SE, Bad Homburg, Germany) ensure a uniform and defined application of plasma and thrombolytic agent. Thus the clot is ensheathed by a mixture of plasma and thrombolytic agent despite the circulating blood substitute. To minimize the hydrodynamic resistance of the carrier, long channels allow the circulating fluid to pass by even if the cavity of the pipe is completely filled with a clot. Channels connected with the cavity by small holes drain more fluid when the clot becomes smaller and the blocked holes open. During clot lysis, the increased flow rate streaming through the cavity-connected-channels leads to a decreased pressure decline measured at the clot carrier.

### Clot preparation

Human venous blood is obtained from different donors of the same blood group who are not taking anticoagulants or antithrombotic medications. Blood sampling has been approved by the ethics committee of the University of Lübeck and is conducted by the local Department of Transfusion Medicine, within the clinical routine. Blood is drawn into citrate tubes (S-Monovette^®^: REF 02.1067.001, Sarstedt, Nümbrecht, Germany) and pooled to avoid individual differences concerning the blood consistency and its quality of clotting. After centrifugation at 180*g *for 10 minutes (Multifuge^® ^1S-R: Heraeus Holding GmbH, Hanau, Germany) 3.5 ml of platelet rich plasma (PRP) are removed by aspiration of the supernatant and mixed with 0.5 ml of the boundary layer, which formed between the supernatant and the erythrocyte layer. Clot formation is achieved by recalcification (final concentration of CaCl_2_: 13.8 mmol/l) and incubation at 37°C for 2 hours.

### Measurement of flow velocity

A custom-built ultrasonic flow meter (developed by the Fraunhofer Institute for Biomedical Engineering IBMT, St. Ingbert, Germany) is used to measure flow velocity based on correlation methods. Two ultrasound transducers are placed directly opposite from each other and aligned parallel to the direction of the streaming fluid (Figure 1). The transducers work alternately as emitter or receiver. Thus, ultrasound can be emitted and received in both directions. In flow direction, the time of flight of the ultrasound (t_+_) is shorter than in the reverse direction (t_-_). Flow velocity is calculated by measuring the time of flight difference of the two emitting directions with a sampling rate of 1 Hz:(1)

### Pressure measurement

Pressure sensors (REF I-99-IA-013, pvb^®^: Critical Care GmbH, Kirchseeon, Germany) are connected to the tube system before (P1) and behind (P2) the clot carrier for a continuous recording of the pressure decline at the clot carrier (ΔP = P1-P2).

### Experimental analysis of the reliability of the model

According to Ohm's law the pressure decline that is measured to monitor thrombolysis depends not only on the clot volume but also on the flow velocity of the streaming blood substitute. Therefore a defined control of the flow velocity is necessary for all experiments with streaming fluids. The model was validated with three different measurements:

1) The accuracy of the flow meter was tested by volumetric measurements. 10 different constant flow velocities were excited by the pump and measured by the flow meter continuously for 5 minutes. The mean of the recorded data was compared with the flow velocity calculated from the collected fluid volume and the cross sectional area of the tube system.

2) The temporal stability of flow velocities excited by the pump was verified by continuous four-hour flow measurements using the ultrasonic flow meter. Two flow velocities were adjusted at the pump, that coincide with the minimum and maximum values of the pulsatile flow velocity used in the subsequent lysis experiments (0.29 and 0.64 m/s). To ensure stable testing conditions the clot carrier was totally filled with non-deformable POM.

3) Furthermore the dependence of flow velocity on the hydrodynamic resistance was analyzed. The pump was set to 5 different flow velocities within the available range of 0 to 0.95 m/s. Flow velocity was recorded for 1 minute in front of the gradually POM-charged clot carrier (0 - 100%) and averaged.

4) Finally the attenuation of ultrasound on its way to the cavity of the clot carrier was determined to test whether it coincides with values found in the literature for the attenuation of diagnostic ultrasound transcranially applied to the M1 segment of the middle cerebral artery of human beings. Ultrasound was supplied by a SONOS 2500 (Hewlett Packard, Palo Alto, California, USA) driven in the transcranial colour-coded ultrasound mode (2 MHz, 0.179 W/cm^2^) as it is commonly used for diagnostic purposes. A custom-built hydrophone (developed by the Fraunhofer Institute for Biomedical Engineering IBMT, St. Ingbert, Germany) with a centre frequency of 2 MHz and a bandwidth of 100% (1 MHz - 3 MHz) was inserted into the centre of the cavity of the clot carrier. According to the experimental setup depicted in Figure 1 the clot carrier was located within the tube system that runs through the basin filled with degassed water. The distance between the hydrophone and the ultrasound transducer was 50 millimetre. Inside the clot carrier the ultrasound intensity I_1 _was determined by the hydrophone and read off an oscilloscope (HP 54520A, Hewlett Packard, Palo Alto, California, USA). For reference measurements the ultrasound intensity I_0 _was determined when ultrasound was applied from the water surface of the basin to the unshielded hydrophone mounted at the same distance of 50 mm within the degassed water. Attenuation A was determined five times by:(2)

### Experimental analysis of the hydrodynamic properties of the custom-built clot carrier

To describe the hydrodynamic properties of the custom-built clot carrier its resistance was calculated by Ohm's law on the basis of pressure declines at the clot carrier at different filling levels and flow velocities of the streaming blood substitute. For each flow velocity and each filling level three independent measurements of the pressure decline were performed. For each measurement the clot carrier was completely disassembled into its component parts and afterwards recomposed. Again, POM was used for the fillings.

### First non-contact and time-continuous measurements of in-vitro thrombolysis

To prove the feasibility of the model the pressure decline at the clot-filled carrier was time-continuously measured for clots solely undergoing a defined pulsatile flow (group A). Flow and pressure were adjusted before the clot carrier was filled to simulate the hydrodynamic situation within the internal carotid artery of a patient with hypertension (Figure 3). Systolic and diastolic flow velocities were set to 0.64 and 0.29 m/s, respectively, leading to a mean pressure (MP) of 130 mmHg.

In a second experimental group B the pressure decline was determined for clots under the same flow conditions and the influence of a typical scenario of sonothrombolysis using diagnostic ultrasound and rt-PA (Actilyse^®^, Boehringer Ingelheim Pharma GmbH & Co. KG, Ingelheim, Germany) as thrombolytic drug. Starting at t = 60 minutes diagnostic ultrasound as described above was continuously applied for 1 hour. The distance between the clot within the clot carrier and the ultrasound transducer was 50 millimetres corresponding to the distance between the M1 segment of the human middle cerebral artery and the acoustic window of the human temporal bone. During ultrasound application 80 ml of a mixture of rt-PA and human blood plasma (final concentration of rt-PA: 60 kU/ml) were continuously injected into the clot carrier. Incubation time for clots of group B was reduced to 1 hour. In both groups A and B the same pulsatile flow was used. For each group 5 experiments with a clot-filled carrier were performed.

A slight shifting of the pressure decline is expected caused by small fluctuations of the flow velocity and temperature of the model. To evaluate this biased error 5 additional measurements with the carrier filled with non-deformable POM were made for each group A and B under the same experimental setups as they were used for the experiments with the clots. These additional measurements serve as control groups. In each experiment, the pressure decline at the clot carrier was continuously measured over a period of 4 hours, using a sampling rate of 10 Hz. Data were processed as follows:

(i = 1, ..., n) is defined to be the pressure decline measured in one of n = 5 experiments of group E = A or B with the carrier filled by a clot (F = CLOT) or POM (F = POM). The biased error D^*E*^(t,V) is determined for each group E by(3)

 is the mean pressure decline of each control group. The biased error is subtracted from each time course measured with a clot under the appropriate experimental condition. For each group A and B this leads to 5 error adjusted time series  of the measured pressure decline:(4)

To allow for a direct comparison between all experiments the "normalized pressure decline"  was defined. It is a dimensionless quantity determined by(5)

At the start of each measurement (t = 0) the value of the normalized pressure decline is 1. When the hydrodynamic resistance of the clot decreases during the measurement the values of  are less than 1 and tend to a small positive value near zero when the clot dissolves completely. The following data of  are reported as mean ± standard error of mean.

## Results

### Reliability of the model

**Figure 4:**
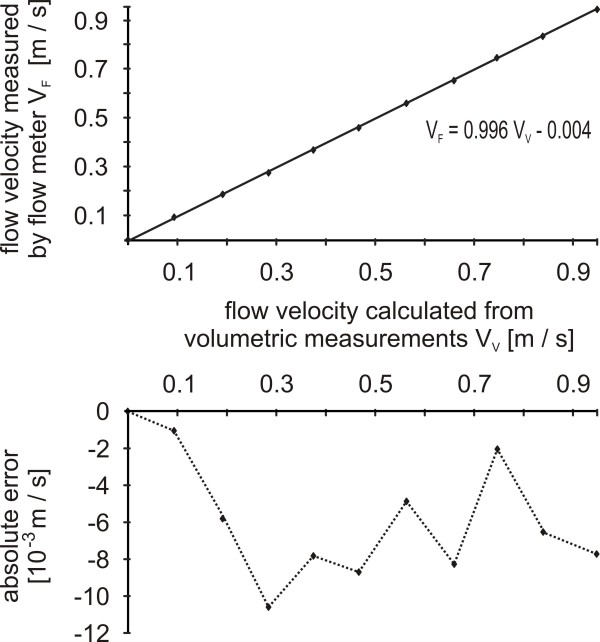
**Accuracy of flow velocity measurement**. Figure above: Comparison between the flow velocities measured by the flow meter (V_F_) and by volumetric measurements (V_V_). Figure below: Absolute error of flow velocity measurements performed by the flow meter.

**Figure 5:**
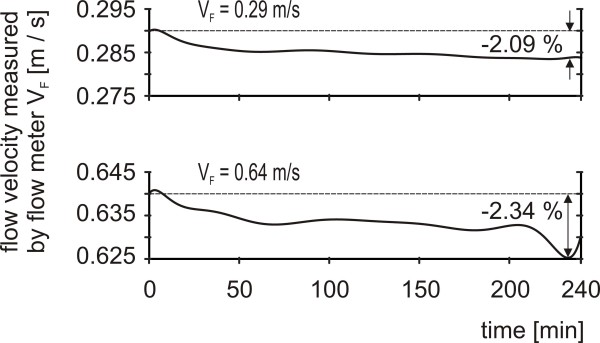
**Temporal stability of flow velocities**. The highest and lowest flow velocity value used in the experiments were adjusted and measured continuously by the flow meter for 4 hours (V_F_).

**Figure 6:**
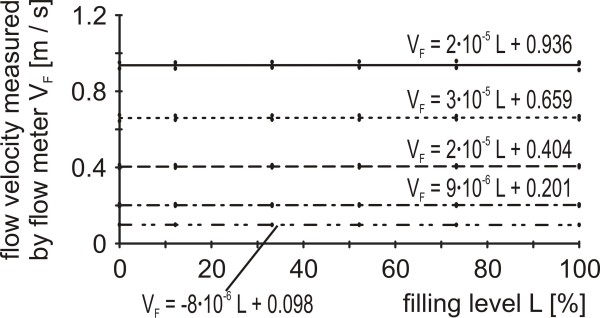
**Stability of flow velocities in dependence of the hydrodynamic resistance**. After adjusting different flow velocities at the pump their values were measured with the flow meter (V_F_) for increasing filling levels (L), respectively hydrodynamic resistances, of the clot carrier.

1) Measuring flow velocity by flow meter lead to marginally lower values compared with results of the volumetric measurement. Within the measuring range of 0 to 0.95 m/s the relative error never exceeded 0.04% (Figure 4).

2) A slight decrease was found for constantly adjusted flow velocities in the course of a four-hour measurement. Its maximum value accounted for 2.34% (Figure 5).

3) Flow velocities generated by the pump varied sparsely with changed hydrodynamic resistance. Within the available flow velocity range of 0 to 0.95 m/s no filling level of the clot carrier caused a relative error beyond 0.81% (Figure 6).

4) For the attenuation of diagnostic 2 MHz-ultrasound (0.179 W/cm^2^) a value of 7.3 dB ± 0.7 dB was found for the described experimental setup.

### Hydrodynamic properties of the custom-built clot carrier

**Figure 7:**
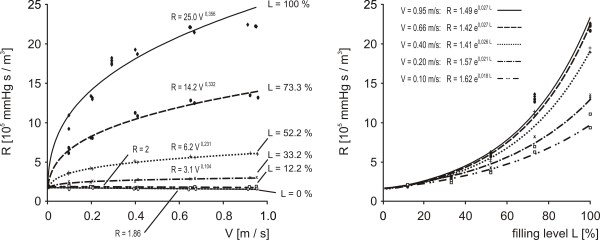
**Calibration curves describing the hydrodynamic behaviour of the clot carrier**. The hydrodynamic resistance of the clot carrier (R) is shown as a function of the flow velocity of the streaming blood substitute (V) and the filling level of the clot carrier (L). For each measurement the experimental setup was completely reassembled to assure totally independent experiments.

At low filling levels the resistance of the clot carrier was constant and independent of the flow velocity of the streaming blood substitute. With increasing filling levels the hydrodynamic resistance of the clot carrier increased exponentially and was correlated by a power law with increasing flow velocities. Repeated measurements showed reproducible results (Figure 7).

### First measurements of in-vitro thrombolysis

**Figure 8:**
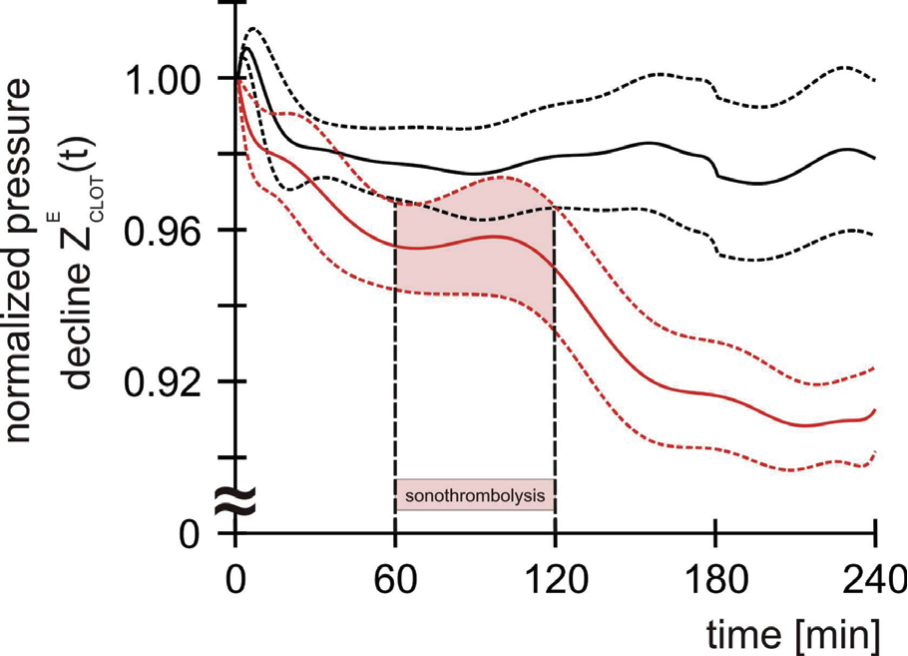
**Normalized pressure decline at the clot-filled carrier for two different thrombolysis experiments (each n = 5)**. Group A (black curves): Clots with an incubation time of 2 hours under a defined pulsatile flow condition. Group B (red curves): Clots with an incubation time of 1 hour under the same pulsatile flow condition undergoing sonothrombolysis (combined treatment with ultrasound and the thrombolytic agent rt-PA) between t = 60 and 120 minutes. Solid lines: Starting point normalized, error adjusted mean values. Dashed lines: Standard errors.

In a four-hour period, no significant difference in thrombolysis between the conditions A and B (Figure 8) was detected. Clots prepared from platelet rich plasma with an incubation time of 2 hours showed only a negligible dissolution under the adjusted pulsatile flow (group A). During the first minutes the normalized pressure decline of group A temporarily increased and stabilized finally at a mean value of about 0.98. Dissolution increased non-significantly in group B. Comparing to group A, clots prepared from platelet rich plasma with an incubation time of only 1 hour dissolved a little bit more during the first hour of the experiment reaching an intermediate value just below 0.96. After treatment with ultrasound and rt-PA the normalized pressure decline further decreased and stabilized at a mean value of about 0.91.

## Discussion

We created an in-vitro pulsatile flow model for the investigation of thrombolysis. In contrast to all previously used experimental setups, the model generates defined physiological or pathological flow conditions. The model allows for time-continuous and non-contact measurements based on the recording of pressure gradients. Compared with other methods of measurement (e.g. magnetic resonance imaging [21], chemical analysis or radiolabeling [14,15,18]), this technique is very inexpensive.

In various experiments the model proved its operational reliability. Flow velocities can be adjusted accurately, remain sufficiently stable for long experimental periods and are nearly independent of the filling levels of the clot carrier. For each measurement of the calibration curve (Figure 7) the experimental setup was completely reassembled to assure totally independent experiments. The collected data prove high reproducibility of the method.

**Figure 9:**
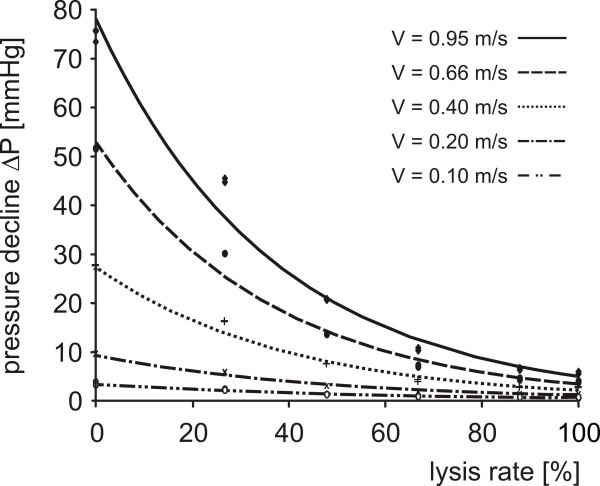
**Rating curves to convert the measured pressure declines into lysis rates**. Every measured pressure decline can be assigned to a lysis rate considering different flow velocities. Thereby pressure declines recorded under different hydrodynamic conditions can be compared. For pulsatile flows the mean velocity should be used. The underlying data are gained by the same measurements as conducted for the calibration curves (Figure 7).

Investigations of the hydrodynamic behaviour of the clot carrier show that clot dissolution can be observed with highest resolution at high filling levels where the highest gradients of the hydrodynamic resistance of the clot carrier are found (Figure 7). Therefore, the model is most suitable for the discrimination of small lysis rates. With increasing filling levels and flow velocities there is an increased eddying flow leading to a non-linear hydrodynamic resistance of the clot carrier. This hydrodynamic behaviour reflects in-vivo turbulences in the region of vessel stenosis and requires an accurate mathematical analysis of the recorded data. For comparing lysis rates achieved under different flow conditions a diagram with rating curves as depicted in Figure 9 is needed.

First experiments concerning the lysis rates of clots prepared from platelet rich plasma reveal only small effects. For group A the hydrodynamic resistance of the clots temporarily increases, probably because the clots are pressed into the carrier by the streaming fluid and a mechanically induced deformation might lead to a tighter occlusion. Although this effect might also occur in group B, the normalized pressure decline decreases, probably caused by the reduced incubation time leading to a lower strength of the less compact clots and an increased attrition at their surfaces. This might also be the reason for the further drop of the normalized pressure decline in group B during the first hour of the experiment. The data do not permit a decision whether the second drop of the normalized pressure decline in group B, starting after approximately 100 minutes, is attributable to those mechanical forces or the impact of sonothrombolysis. Therefore higher lysis rates have to be achieved, which would enable the discrimination between experiments with or without the application of sonothrombolysis. The small lysis rates found in the reported experiments are presumably caused by the following reasons:

1. Thrombolysis is mainly affected by the composition of the used clots [24]. The platelet rich clots of the current experiments are of a very firm and robust consistence with a relative clot mass loss of about 17% after a 2-hour incubation at 37°C in a solution of buffered plasma with pH = 7.4 and rt-PA (final concentration: 60 kU/ml) [28]. According to Mizushige et al. [29] who reported on low lysis rates for artificial white thrombi, this is low compared to the mass loss of about 64% we found under the same treatment for clots generated by spontaneously clotted whole venous blood [28]. Such clots are used by many other research groups for experiments with static or non-physiological flow conditions [4,11]. On the other hand, spontaneously clotted whole venous blood is unsuited for experiments under physiological flow conditions because it completely dissolves within seconds under the influence of the streaming fluid [28]. This work was not focused to investigate the clotting process in detail but the results point out that for future experiments clots are needed that resist pulsatile flows on the one hand, and show sufficient lysis rates on the other hand. In our opinion, the low lysis rates of the first experiments with platelet rich clots reflect those clinical situations, in which delayed, incomplete, or even no lysis are observed [30].

2) It might be possible, that the used amount of rt-PA and plasma is too small for a sufficient thrombolysis. Therefore dose-finding experiments are planned.

3) Finally attenuation of the applied ultrasound might be too high. Measurements revealed a value of about 7.3 dB for the used diagnostic 2 MHz-ultrasound (0.179 W/cm^2^) in the described experimental setup. Aaslid et al. report on a value of 7.0 dB for a 2 MHz-ultrasound passing through the human skull [31]. Pfaffenberger et al. determined ultrasound attenuations between 8.8 and 21.2 dB using 5 temporal bones of different thickness and a diagnostic ultrasound device (Sonos 4500, Philips, Hamburg, Germany) with a mean frequency of 1.8 MHz [10]. Although these data imply that attenuation measured in the experimental setup has the appropriate physiological dimension, it has to be considered that measurements carried out with different ultrasound devices can not be compared directly.

Despite low lysis rates the essential advantage of the presented model is clearly evident: Unlike all previously used models the dynamic process of thrombolysis can now be observed online and time-continuously without touching the clot and interfering with the process of lysis. As any model of physiological systems, the method includes also certain simplifications:

1. The complex function of the endothelium is not considered and blood has to be replaced by a substitute to avoid uncontrollable coagulation. Interactions between blood and artificial surfaces of the model will inevitably lead to unforeseeable changes of coagulation and fibrinolysis [32] just like the use of anticoagulated blood [33]. To avoid those complications we decided to use a Ringer solution as circulating blood substitute and to inject plasma directly into the clot carrier during the process of sonothrombolysis. Thereby, we hope to create a physiological microenvironment of plasma ensheathing the clot. Compared with blood, the Ringer solution we used has a lower viscosity. If the measured effects were attributable to merely the mechanical properties of the used fluid, however, they should be more pronounced in vivo.

2. Measurement errors caused by slight changes of the flow velocity and temperature of the model vary with the chosen hydrodynamic condition. We calculated an error adjusted pressure decline for an individual correction of each modelled hydrodynamic condition. From a mathematical point of view this is not absolutely correct because different measurements were offset against each other.

3. Even the empty clot carrier has a measurable hydrodynamic resistance. Therefore the defined normalized pressure decline that always starts with the value 1 can never reach value 0. This leads to problems when high lysis rates of experiments with different flow velocities are compared, because the final value attained for complete clot dissolution varies in dependence of the adjusted flow velocity. In such cases the normalized pressure decline has to be replaced by conversions based on the measured rating curves (Figure 9).

4. The presented experiments can not differentiate between mechanical deformation and enzymatic lysis of the clots. In future the D-dimer level in the streaming fluid will be determined as a marker for enzymatic fibrinolysis to overcome this inaccuracy.

## Conclusion

Despite all mentioned limitations, we believe that our model is suitable for investigating the effects of mechanical and biochemical forces on clot dissolution. Thrombolysis is monitored by a new technique that is based on the measurement of the pressure decline at the clot carrier. The model enables the assessment of physiological flow conditions and non-contact, time-continuous measurements without disturbing the process of thrombolysis, as well as a defined adjustability of parameters and accessibility for thrombolytics and ultrasound.

The temporal effect of commonly used lysis strategies can now be investigated with high resolution. The online presentation of the measured data allow for an interactive and precise change of the model parameter even during the course of an experiment. Therefore the model provides ideal conditions for investigations of the mode of action of sonothrombolysis and will be a powerful tool in finding the most effective lysis strategy.

## Competing interests

The authors had full access to the data and take responsibility for its integrity. All authors have read and agree to the manuscript as written. There are no conflicts of interest.

## Authors' contributions

FCR designed the model, carried out the experiments, analyzed and interpreted the data and wrote the manuscript. MO assisted in doing the experiments and made substantial contributions to acquisition of data. JHM and FS were substantially involved in all questions concerning the clot preparation. MS and PKW provided technical equipment and support. PT made substantial contributions to analysis and interpretation of data. JE made substantial contributions to interpretation of data. GS made important contributions to the model design and interpretation of data. All authors revised the manuscript critically and approved its final version.

## Pre-publication history

The pre-publication history for this paper can be accessed here:

http://www.biomedcentral.com/1471-2377/11/58/prepub
